# Individuating Logics: A Category‐Theoretic Approach

**DOI:** 10.1002/tht3.425

**Published:** 2019-06-18

**Authors:** John Wigglesworth

**Affiliations:** ^1^ University of Vienna

**Keywords:** philosophy of logic, theoretical equivalence, anti‐exceptionalism, categorical equivalence, institutions

## Abstract

This paper addresses a recent debate as to whether logical anti‐exceptionalists should understand logical theories in syntactic or semantic terms. In Wigglesworth (2017), I propose a purely semantic approach, while Woods (2018) has argued in favor of a purely syntactic approach. Here, I argue that neither of these approaches is satisfactory, as both treat arguably distinct logics as equivalent logical theories. I argue instead for an approach that combines syntactic and semantic components. The specific approach to a combined account of logical theories is based on the category‐theoretic notion of an institution.

## Subject area: Philosophy of logic

In Wigglesworth (2017), I argue against a purely syntactic conception of logical theories. The argument proceeds by showing that a plausible syntactic account of theoretical equivalence, when applied to logical theories, has the unfortunate consequence that it makes classical and intuitionistic logic equivalent. Based on this argument, I suggested a purely semantic conception of logical theories, understanding them in terms of categories of models. In response, Jack Woods (2018) has proposed a stronger syntactic account of theoretical equivalence, according to which classical and intuitionistic logic are not equivalent. Based on this proposal, Woods argues that a purely syntactic account of logical theories remains plausible. In this note, I show that both purely syntactic accounts and purely semantic accounts are subject to counterexamples that bring them into question. I then propose an account that combines syntactic and semantic components, which avoids these counterexamples. The specific approach to a combined account of logical theories is based on the category‐theoretic notion of an institution.

To summarize the arguments in Wigglesworth (2017) and Woods (2018), take a theory to be a set of sentences closed under a relation of logical consequence. Given a set of sentences, *S*, the theory that *S* generates by closing *S* under a logic ℒ is the set $T_{S}^{ℒ}$. With this definition in hand, there is a natural syntactic criterion for the equivalence of logical theories. The syntactic criterion invokes the notion of a mapping, *τ*, from the language of one theory, *T*, to the language of another, *T* ^*′*^. The mapping must satisfy certain requirements. In the context of logical theories, it should at least commute with the negation operator, so that *τ*(¬*φ*) := ¬*τ*(*φ*). The mapping *τ* is then a translation from *T* to *T* ^*′*^ iff for all *φ* ∈ *T*, *τ*(*φ*) ∈ *T* ^*′*^. The theories *T* and *T* ^*′*^ are intertranslatable iff there are translations *τ* : *T* → *T* ^*′*^, and *σ* : *T* ^*′*^ → *T*. Two logics, ℒ_1_ and ℒ_2_ are then syntactically equivalent iff for all sentences *S*, $T_{S}^{{ℒ}_{1}}$ is intertranslatable with $T_{S}^{{ℒ}_{2}}$.

The argument in Wigglesworth (2017) showed that, under this syntactic criterion, classical and intuitionistic logic are equivalent, as for any set of sentences, *S*, the classical theory of *S* is intertranslatable with the intuitionistic theory of *S*. Translations between the classical and intuitionistic theories of any *S* are given, in one direction by the Gödel‐Gentzen translation from the classical theory to the intuitionistic theory, and in the other direction by the identity mapping. As classical and intuitionistic logic should not be equivalent logical theories, I argued that purely syntactic approaches to theoretical equivalence are inappropriate for logical theories. I suggested that a semantic approach might yield better results in this particular case, and I considered an account of theoretical equivalence given in terms of categories of models. According to this semantic account, one can show that classical and intuitionistic logic are not equivalent, as they shouldn't be.

Woods (2018) argues that there is a stronger syntactic criterion that is able to distinguish between classical and intuitionistic logic. The stronger criterion is achieved by making three refinements to the intertranslatability account.^1^ Recall that the current intertranslatability account determines two logical theories, ℒ_1_ and ℒ_2_, to be syntactically equivalent when, for all *S*, there are translations $\tau :T_{S}^{{ℒ}_{1}}\to T_{S}^{{ℒ}_{2}}$ and $\sigma :T_{S}^{{ℒ}_{2}}\to T_{S}^{{ℒ}_{1}}$.

The first refinement to this account allows for comparisons between logics with different logical expressions. For example, we might want to compare theories formulated in the language of disjunction, negation, and True with theories formulated in the language of conjunction, negation, and False. Or we might want to compare notational variations of a given logical theory, for example, one that uses the standard interpretations of ∧ and ∨ and one that treats ∧ as disjunction and ∨ as conjunction. Though my original presentation of the intertranslatability account does not explicitly rule out these comparisons, more must be said about how they would work. To apply the equivalence conditions in cases like these, one can take translations to be relative to mappings between the logical expressions of the relevant languages. Let *t* map the logical expressions of ℒ_1_ to those of ℒ_2_, and let *t*(*S*) be the result of applying this mapping to the sentences in *S*. Let *u* be a mapping in the other direction. The first refinement then results in the following proposal for syntactic equivalence: Two logical theories, ℒ_1_ and ℒ_2_, are syntactically equivalent when, for all *S*, there are translations $\tau :T_{S}^{{ℒ}_{1}}\to T_{t\left(S\right)}^{{ℒ}_{2}}$ and $\sigma :T_{S}^{{ℒ}_{2}}\to T_{u\left(S\right)}^{{ℒ}_{1}}$.

The second refinement rules out different translations for different sets *S*. The intertranslatability criterion for syntactic equivalence between two logics, ℒ_1_ and ℒ_2_, requires that for any *S*, there is some translation from the theory that *S* generates according to ℒ_1_ to the theory that *S* generates according to ℒ_2_. But the translation can be local to *S*, and each *S* can use a different translation. The second refinement requires general translations, such that each *S* uses the same translation mapping. Incorporating this refinement, the proposal for syntactic equivalence becomes: Two logical theories, ℒ_1_ and ℒ_2_, are syntactically equivalent when there are translations *τ* and *σ* such that for all $S,\tau :T_{S}^{{ℒ}_{1}}\to T_{t\left(S\right)}^{{ℒ}_{2}}$ and $\sigma :T_{S}^{{ℒ}_{2}}\to T_{u\left(S\right)}^{{ℒ}_{1}}$.

So far, the refinements that Woods proposes do not block the translations between classical and intuitionistic logic that provide a counterexample to the intertranslatability criterion for theoretical equivalence between logics. Classical logic and intuitionistic logic have the same logical expressions, and the relevant translations can be applied to every set *S*. Woods proposes a third refinement, however, which is meant to rule out these “perverse” translations. Given two logics, ℒ_1_ and ℒ_2_, this refinement requires that when translating a sentence *φ* of the language of ℒ_1_ to a sentence *τ*(*φ*) of the language of ℒ_2_, the sentences *φ* and *τ*(*φ*) should be logically equivalent according to the logic ℒ_2_.

In arguing against the intertranslatability account, I used the Gödel‐Gentzen translation to provide a mapping, *γ*, for any *S*, from the classical theory of *S* to the intuitionistic theory of *S*. This translation does not satisfy the third refinement. For example, *γ* maps *p* ∨ ¬*p* to ¬(¬*p* ∧ ¬¬*p*), and these are not intuitionistically equivalent. Formally, Woods incorporates the third refinement into a syntactic account of theoretical equivalence as follows: Two logical theories, ℒ_1_ and ℒ_2_, are syntactically equivalent, relative to the mappings, *t* and *u*, between logical expressions, when there are general translations *τ* and *σ* as before. Additionally, for any *S* in the language of ℒ_1_, the theory generated by *S* according to ${ℒ}_{1},T_{S}^{{ℒ}_{1}}$, must be ℒ_1_‐equivalent to the theory one gets by translating the theory generated by *S* according to ℒ_2_ into the language of ℒ_1_, $\sigma \left(T_{t\left(S\right)}^{{ℒ}_{2}}\right)$. The analogous equivalence according to ℒ_2_ must hold for any *S* in the language of ℒ_2_. Theories $T_{S}^{{ℒ}_{1}}$ and $\sigma \left(T_{t\left(S\right)}^{{ℒ}_{2}}\right)$ are ℒ_1_‐equivalent when for every sentence $\psi \in T_{S}^{{ℒ}_{1}}$, there is a sentence $\varphi \in \sigma \left(T_{t\left(S\right)}^{{ℒ}_{2}}\right)$ such that $T_{\left\{\varphi \right\}}^{{ℒ}_{1}}=T_{\left\{\psi \right\}}^{{ℒ}_{1}}$.

It may be that the refined syntactic account of theoretical equivalence can distinguish between classical and intuitionistic logic. However, one can show that there are other intuitively distinct logical theories that Woods' proposal judges to be theoretically equivalent. That is, there are logical theories that are arguably distinct, and yet there are translations between them that satisfy these stronger syntactic conditions. The most obvious cases are distinct logical theories that make exactly the same inferences valid. A well‐known example compares classical logic and supervaluationist logic. Supervaluationist logic is nonclassical, because it allows for sentences to be assigned a truth value other than True or False. This feature of supervaluationist logic makes it an attractive formal framework to capture reasoning with nonclassical concepts, like vague concepts (Williamson 1994) and the concept of truth (Burgess 1986; McGee 1991). Sentences that describe these nonclassical concepts can take an intermediate truth value, and the semantics for supervaluationist logic describe how this nonclassical truth value works.

Supervaluationist semantics is based on strong Kleene logic, a three‐valued logic whose third value, *i*, is usually interpreted as *indeterminate* or *neither true nor false*.^2^ Strong Kleene logic defines consequence in the standard way, as preservation of truth across all models. Supervaluationist logic has a more nuanced account of consequence, which is given in terms of supervaluations. Take any strong Kleene valuation, *v*, which may assign some sentences the third truth value *i*. Let *v* ≤ *v′* mean that *v′* is a classical valuation which agrees with the classical truth values that *v* assigns to sentences. The classical valuation *v′* is called a *precisification* or *resolution* of *v*, as it resolves any of the indeterminacies in *v*. For any sentence *φ* that is not assigned True or False under *v* (and so receives the third value *i*), *v′* assigns *φ* a classical truth value. Let the supervaluation of a sentence *φ* be a map *v* ^+^, where

*v* ^+^(*φ*) = True iff for all *v′*, such that *v* ≤ *v′*, *v′*(*φ*) = True.

*v* ^+^(*φ*) = False iff for all *v′*, such that *v* ≤ *v′*, *v′*(*φ*) = False.

*v* ^+^(*φ*) = *i* otherwise.

Validity in supervaluationist logic can then be defined as follows:

$S{⊨}_{\mathrm{SV}}\psi$ if and only if for every valuation *v*, if *v* ^+^(*φ*) = True for all *φ* ∈ *S*, then *v* ^+^(*ψ*) = True.

Interestingly, while supervaluationist logic is used to capture reasoning with nonclassical concepts, one can show that for any *S* and $\psi ,S{⊨}_{\mathrm{SV}}\psi$ if and only if *ψ* is a classical consequence of *S*. From the point of view of the syntactic approach to logical theories, supervaluationist logic is identical to classical logic.

It follows that for any set of sentences, *S*, the classical theory of *S* is intertranslatable with the supervaluationist theory of *S*, because the theories are identical: one can apply the identity translation in both directions. Furthermore, the identity translation satisfies the three refinements that Woods proposes. Classical logic and supervaluationist logic use the same logical expressions; the identity translation is a general translation that can be applied to any *S*; and the identity translation is not perverse, as the resulting theories are identical and therefore logically equivalent according to both classical and supervaluationist logic.

What the example of classical and supervaluationist logic shows is that there are logics that are intuitively distinct, but which satisfy very strong notions of syntactic equivalence.^3^ This suggests that a purely syntactic approach cannot distinguish between some intuitively distinct logics.

Unfortunately, it is apparent that a purely semantic approach cannot do this either. The semantic approach that I proposed in Wigglesworth (2017, p. 764) is adopted from a standard account of semantic equivalence that one finds in the philosophy of science, an account that appeals to category theory (see Halvorson 2016; Weatherall 2016). Consider the theories $T_{S}^{{ℒ}_{1}}$ and $T_{S}^{{ℒ}_{2}}$, generated by a set of sentences, *S*, according to the logics ℒ_1_ and ℒ_2_. Let **Mod** ${}_{{ℒ}_{1}}\;$(*S*) be the category of models of the theory $T_{S}^{{ℒ}_{1}}$, where the objects of **Mod** ${}_{{ℒ}_{1}}\;$(*S*) are the models of *S* as given by the standard model theory for ℒ_1_, and the arrows are homomorphisms between models.^4^ Similarly for the category **Mod** ${}_{{ℒ}_{2}}$(*S*). These categories are equivalent if and only if there are functors *F*: **Mod** ${}_{{ℒ}_{1}}\;$(*S*) → **Mod** ${}_{{ℒ}_{2}}\;$(*S*) and *G*: **Mod** ${}_{{ℒ}_{2}}\;$(*S*) → **Mod** ${}_{{ℒ}_{1}}\;$(*S*) such that $\mathrm{GF}≅1_{{\mathrm{Mod}}_{{ℒ}_{1}}\left(S\right)}$ and $\mathrm{FG}≅1_{{\mathrm{Mod}}_{{ℒ}_{2}}\left(S\right)}$. That is, the categories are equivalent when there are functors between them whose compositions are naturally isomorphic to the identity functors of the respective categories. I suggested that two logical theories, ℒ_1_ and ℒ_2_, are semantically equivalent if and only if for all sentences *S*, the category of models of *S*, as given by the model theory of ℒ_1_, is equivalent (in the category‐theoretic sense) to the category of models of *S*, as given by the model theory of ℒ_2_. One can then show that on this semantic account, classical and intuitionistic logic are not equivalent.

Unfortunately, this purely semantic criterion does not generalize to cover all cases. There are logical theories that satisfy this category‐theoretic condition for equivalence, but which are intuitively distinct. The most obvious cases are logics that appeal to the same category of models in order to develop their model theory. One example of this is given by supervaluationist logic, or more precisely, by two approaches to supervaluationist logic.

Supervaluationism comes in several varieties, depending on how key notions are defined.^5^ One of those notions is logical consequence. Above we defined supervaluationist consequence as:

$S{⊨}_{\mathrm{SV}}\psi$ if and only if for every valuation *v*, if *v* ^+^(*φ*) = True for all *φ* ∈ *S*, then *v* ^+^(*ψ*) = True.

Following Williamson's (1994) terminology, this definition gives us a *global* notion of supervaluationist consequence. One can also define a *local* notion of supervaluationist consequence:

$S{⊨}_{\mathrm{SV}}\psi$ if and only if for every valuation *v*, for every precisification *v′* of *v*, if *v′*(*φ*) = True for all *φ* ∈ *S*, then *v′*(*ψ*) = True.

In the single‐conclusion case, the local and global definitions of consequence are equivalent. However, in the multiple‐conclusion case, there are differences.

A multiple‐conclusion inference *S* ⊨ *R* is a relation between two sets of sentences, where *R* may contain more than one sentence. The intuitive idea is that a multiple‐conclusion inference is valid iff every model that makes all of the sentences in *S* true makes some sentence in *R* true. Defining consequence in multiple‐conclusion terms gives us the following variations on global and local supervaluationism.

$S{⊨}_{\mathrm{SV}}R$ if and only if for every valuation *v*, if *v* ^+^(*φ*) = True for all *φ* ∈ *S*, then *v* ^+^(*ψ*) = True for some *ψ* ∈ *R*.

$S{⊨}_{\mathrm{SV}}R$ if and only if for every valuation *v*, for every precisification *v′* of *v*, if *v′*(*φ*) = True for all *φ* ∈ *S*, then *v′*(*ψ*) = True for some *ψ* ∈ *R*.

These definitions of supervaluationist consequence do not match up, as there are multiple‐conclusion inferences that are valid according to the second definition but invalid according to the first. For example, the inference $\varphi \vee \neg \varphi {⊨}_{\mathrm{SV}}\varphi ,\neg \varphi$ is locally valid but globally invalid. Consider the valuation that assigns *φ* the third truth value *i*, so that ¬*φ* is also *i*, but *φ* ∨¬*φ* is True.

Here, we have two different logics (because they make different inferences valid) given by two different definitions of logical consequence over the same collection of models. Unfortunately for the purely semantic approach, there is no way to capture this difference in the category‐theoretic framework given above. Both local and global supervaluationism use exactly the same category of models to assign truth values to sentences. It follows that, for any set of sentences *S*, the category of local supervaluationist models and the category of global supervaluationist models are more than equivalent (in the category‐theoretic sense); they're identical. This standard semantic criterion for theoretical equivalence, given in terms of category theory, therefore judges local and global supervaluationism to be equivalent, when intuitively they should not be.

The difference between local and global supervaluationist logic is given by how they define logical consequence. Unfortunately, because categorical equivalence only focuses on models as objects, and on homomorphisms between models, the distinction between local and global supervaluationism, given in terms of the definition of consequence, is lost. This problem will generalize to any distinct logics given by different definitions of logical consequence over the same category of models.

To take an example that does not require multiple‐conclusion inferences, consider strong Kleene logic and Graham Priest's (1979) logic of paradox, ℒP. Both logics, in their single‐ and multiple‐conclusion versions, define logical consequence over the same category of models. The logics differ with respect to their designated truth‐values, the truth‐values that must be preserved for an inference to be valid. Strong Kleene requires the preservation of truth from the premises to the conclusion, while ℒP requires that if all the premises are true or take the third value *i*, then the conclusion must be true or take the third value *i*. This difference results in the logics making different inferences valid. For example, *modus ponens*, the inference from *φ* and *φ* → *ψ* to *ψ* is valid in strong Kleene but invalid in ℒP. Unfortunately, as before, there doesn't seem to be any way to capture this difference in the category‐theoretic framework. What these cases show is that there are logics that are intuitively distinct, but which satisfy very strong notions of semantic equivalence. This suggests that a purely semantic approach cannot distinguish between some intuitively distinct logics.

As there appear to be counterexamples to both purely syntactic and purely semantic accounts of theoretical equivalence, it is likely that in the case of logical theories, it may be necessary to combine syntactic and semantic components. One option to combine syntax and semantics invokes the category‐theoretic notion of an institution.

An institution is an ordered tuple ℐ = ⟨**Sig**, Sen, Mod, ⊨⟩. **Sig** is a category of signatures, or languages; Sen is a functor from the category **Sig** to the category **Set** of sets; Mod is a (contravariant) functor from **Sig** to the category **Cat** of categories; and ⊨ is a collection of satisfaction relations ⊨_∑_, one for every signature ∑ in **Sig**. Institutions combine syntactic and semantic components by building in relationships between formal languages (given by **Sig**) and models (given by Mod), such as the relationships explicitly given by the satisfaction relations ⊨_∑_.

A logic, or a logical theory, can be understood as an institution. Each component of an institution is relatively straightforward. Because institutions are supposed to be quite abstract and flexible, they allow for logics in which the signature can vary. For this reason, **Sig** can contain multiple distinct signatures. The morphisms of **Sig** can be given by any function between signatures, as long as it satisfies the usual category‐theoretic conditions on morphisms (the existence of identity morphisms, the composition of morphisms, etc.). For each signature ∑ in **Sig**, Sen (∑) gives us the ∑‐sentences, the well‐formed formulas of the signature; Mod(∑) gives us the models of ∑; and ⊨_∑_ captures the model‐theoretic notion of satisfaction that holds between models and sentences. Logical consequence is then given semantically, in terms of the sentences that models satisfy.

Institutions are able to capture an incredibly wide range of logics, including classical propositional logic, classical first‐order logic, higher order logics, nonclassical logics like intuitionistic logic, modal logics, and many‐valued logics like strong Kleene and supervaluationist logic, fuzzy logics. A particular logic is given by defining the models and the satisfaction relation. For example, for classical propositional logic, the models of a signature, Mod(∑), comprise functions from ∑ to the set {0, 1}. For classical first‐order logic, the models are Tarskian models. In both cases, satisfaction is given in the usual way. For intuitionistic logic, the models are Kripke structures, and satisfaction is possible‐worlds satisfaction.

Not only can institutions capture a wide range of logics, one can also define an equivalence relation on institutions. An equivalence between two institutions, ℐ and J, comprises a comorphism from ℐ to J that satisfies certain conditions. A comorphism $\left(\Phi ,\alpha ,\beta \right):ℐ\to J$ includes a functor, Φ, from **Sig** ^ℐ^ to **Sig** ${}^{J}$, a natural transformation, *α*, from Sen ^ℐ^ to ${\text{Sen}}^{J}\circ \;\Phi$, and a natural transformation, *β*, from ${\text{Mod}}^{J}\circ \;\Phi^{\mathrm{op}}$ to Mod ^ℐ^. Additionally, the following satisfaction condition must hold. For each signature ∑ in **Sig**, each model *M* in ${\mathrm{Mod}}^{J}\left(\Phi \left(\sum \right)\right)$, and each sentence *φ* in Sen ^ℐ^(∑):

$$M{⊨}_{\Phi \left(\sum \right)}^{J}\alpha_{\sum }\left(\varphi \right)\;\mathrm{iff}\;\beta_{\sum }\left(M\right){⊨}_{\sum }^{ℐ}\varphi$$

Essentially, this condition requires that *M* satisfies the image of *φ* under the transformation *α* if and only if the image of *M* under the transformation *β* satisfies *φ*.

Equivalence between two institutions is then defined as the existence of a comorphism between them that satisfies all of the following conditions:

Φ is an equivalence of categories,
*α*_∑_ has an inverse, up to semantic equivalence, which is natural in ∑, and
*β*_∑_ is an equivalence of categories.^6^

This relation of institutional equivalence matches our intuitions about the equivalence of logical theories in many cases. For example, classical propositional and first‐order logic are not equivalent to their intuitionistic counterparts (Mossakowski et al. 2007, Example 4.9). Using institutions for many‐valued logics, one can also distinguish between classical, supervaluationist, and strong Kleene logics (Diaconescu 2013). So, the notion of institutional equivalence gets it right in cases where we want to say certain logics are not equivalent.

It also gets it right in cases where we want to say certain logics are equivalent. For example, we would like different presentations of classical propositional logic, for example, those that take different sets of logical expressions as primitive, to be equivalent logical theories. Treating logics as institutions can do this. One can show that classical propositional logic given in terms of disjunction, negation, and True, when understood as in institution, is equivalent to classical propositional logic given in terms of conjunction, negation, and False (Mossakowski et al. 2007, Example 3.9).

Given these successes, understanding logical theories in terms of institutions is an attractive alternative that combines syntactic and semantic components. Institutions give us a unified approach that gets it right in cases where purely syntactic and purely semantics approaches fail. The arguments presented in Wigglesworth (2017) and Woods (2018) are given in the context of a view known as logical anti‐exceptionalism. Anti‐exceptionalism about logic takes logical theories to be continuous with scientific theories. If one has this view of logical theories, it is reasonable to examine them with respect to debates in the philosophy of science that focus on the structure of scientific theories. These debates have concentrated on two approaches: the so‐called received view, which takes a purely syntactic approach, and the semantic view, which takes a purely semantic approach. These two approaches diverge considerably in their understanding of the structure of scientific theories, and the debate as to which approach is preferable continues to be a lively one. However, in the special case of logical theories, the above considerations suggest that an approach which combines syntactic and semantic components is to be preferred. The formal, category‐theoretic notion of an institution, coupled with a suitable equivalence relation on institutions, offers a combined approach that matches many of our intuitions about which logical theories are and are not equivalent. These results make a strong case for the approach that understands logical theories in terms of institutions.

## Notes

1 Similar refinements are independently proposed in Dewar (2018).

2 For more details, see Priest (2008), chs. 7 and 21.

3 This case also provides a counterexample to the syntactic approach proposed by Dewar (2018).

4 One may also use elementary embeddings as arrows.

5 See Varzi (2007) for a nice discussion of different approaches to supervaluationism.

6 Further details can be found in Diaconescu (2008), p. 41, and Mossakowski et al. (2007), Definition 3.5.
